# Supplementary material for: Social experience shapes fighting strategies in Drosophila
Source: eLife. 2025 Oct 7;13:RP104212. doi: 10.7554/eLife.104212 (PMC12503488; doi:10.7554/eLife.104212)
Supplement: Supplementary file 1. [file elife-104212-supp1.docx]

**Supplementary File 1. Detailed information for fly stocks.**

| **Figures** | **Genotypes** | **Housing conditions** | **Sample sizes** |
| --- | --- | --- | --- |
| Figure 1 | | | |
| B | *Canton-S* | GH-2d,  GH-7d,  GH-14d | n=30, 30, 30, 46, 46, 46 |
| C | *Canton-S* | GH-14d | n=30 for each group |
| D | *Canton-S* | GH-14d | n=30 for each group |
| Figure 2 | | | |
| B | *Canton-S* | SH-14d,  GH-14d |  |
| C, D, E, F | *Canton-S* | SH-14d,  GH-14d | n=27 for SH, n=30 for GH |
| G | *Canton-S* | SH-14d,  GH-14d | n=30 events from 10 samples for each group |
| Figure 3 | | | |
| A, B, C, D | *w+;;UAS-Kir2.1/+* | GH-14d | n=30 for A, B; n=18 for C, D |
|  | *w+;;Or67d-GAL4/UAS-Kir2.1* | GH-14d |  |
|  | *w+;Or65a-GAL4/+;UAS-Kir2.1/+* | GH-14d |  |
|  | *w+;;Or47b-GAL4/UAS-Kir2.1* | GH-14d |  |
|  | *w+;;Ir84a-GAL4/UAS-Kir2.1* | GH-14d |  |
| E, F | *w+;;Or47b-GAL4/+* | GH-14d | 30 |
|  | *w+;;Or47b-GAL4/UAS-Or47b-RNAi* | GH-14d | 30 |
|  | *w+;;Or47b-GAL4/UAS-fruMi* | GH-14d | 30 |
| G, H | *w+;;Or47b-GAL4/+* | GH-14d | 60 |
|  | *w+;;Or47b-GAL4/UAS-NaChBac* | GH-14d | 60 |
|  | *w+;;Or47b-GAL4/+* | SH-14d | 60 |
|  | *w+;;Or47b-GAL4/UAS-NaChBac* | SH-14d | 49 |
| Figure 4 | | | |
| A, B | *w+;;UAS-Kir2.1/+* | GH-14d | 30 |
|  | *w+;;R19B03-GAL4/UAS-Kir2.1* | GH-14d | 30 |
|  | *w+;R15A01-AD/+;R71G01-DBD/UAS-Kir2.1* (*P1^a^ > UAS-Kir2.1*) | GH-14d | 30 |
|  | *w+;;TK-GAL4/UAS-Kir2.1* (GH-14d) | GH-14d | 30 |
|  | *w+;VT002064-AD/+;VT008469DBD/UAS-Kir2.1* (*pC1^SS1^ > UAS-Kir2.1*) | GH-14d | 30 |
|  | *w+;;VT002064-AD,dsxDBD/UAS-Kir2.1* (*pC1^SS2^ > UAS-Kir2.1*) | GH-14d | 30 |
| C | *w-;R15A01-AD/UAS-myrGFP; R71G01-DBD/+* (*P1^a^ > UAS-GFP*) | GH-5-7d |  |
| D | *w-;UAS-myrGFP/+;VT002064-AD,dsxDBD/+* (*pC1^SS2^ > UAS-GFP*) | GH-5-7d |  |
| F, G, H | *w-;;UAS-CsChrimson/+* | GH-5-7d | 32 |
| I, J, K | *w-;R15A01-AD/+;R71G01-DBD/ UAS-CsChrimson* (*P1^a^ > UAS-CsChrimson*) | GH-5-7d | 32 |
| L, M, N | *w-;;VT002064-AD,dsxDBD/UAS-CsChrimson* (*pC1^SS2^ > UAS-CsChrimson*) | GH-5-7d | 32 |
| Figure 5 | | | |
| A, B, C, D | *w-;R15A01-AD/UAS-myrGFP; R71G01-DBD/+* (*P1^a^ > UAS-GFP*) | GH-5-7d | n=5 for each group (B)  n=9 for each group (D) |
|  | *w-;UAS-myrGFP/+;VT002064-p65AD,dsxDBD/+* (*pC1^SS2^ > UAS-GFP*) | GH-5-7d |  |
| E | *w-;UAS-CsChrimson/+;VT002064-AD, dsxDBD/+* (*pC1^SS2^ > UAS-CsChrimson*) | GH-5-7d |  |
| F | *w-;UAS-CsChrimson/+; VT002064-AD, dsxDBD/UAS-dsx^M^-RNAi* (*pC1^SS2^ > UAS-CsChrimson & UAS-dsx^M^-RNAi*) | GH-5-7d |  |
| G | *w-;UAS-CsChrimson/+;VT002064-AD, dsxDBD/+* (*pC1^SS2^ > UAS-CsChrimson*) | GH-5-7d | 10 |
|  | *w-;UAS-CsChrimson/+;VT002064-AD, dsxDBD/UAS-dsx^M^-RNAi* (*pC1^SS2^ > UAS-CsChrimson & UAS-dsx^M^-RNAi*) | GH-5-7d | 9 |
| H | *w-;UAS-CsChrimson/+;VT002064-AD, dsxDBD/+* (*pC1^SS2^ > UAS-CsChrimson*) | GH-5-7d | 10 |
|  | *w-;UAS-CSChrimson/+;VT002064-AD, dsxDBD/UAS-dsx^M^-RNAi* (*pC1^SS2^ > UAS-CsChrimson & UAS-dsx^M^-RNAi*) | GH-5-7d | 10 |
| I | *w-;UAS-CsChrimson/+;VT002064-AD, dsxDBD/+* (*pC1^SS2^ > UAS-CsChrimson*) | GH-5-7d | 12 |
| J | *w-;UAS-CsChrimson/+;VT002064-AD, dsxDBD/UAS-dsx^M^-RNAi* (*pC1^SS2^ > UAS-CsChrimson & UAS-dsx^M^-RNAi*) | GH-5-7d | 12 |
| K | *w-;UAS-CsChrimson/+;VT002064-AD, dsxDBD/+* (*pC1^SS2^ > UAS-CsChrimson*) | GH-5-7d | 12 |
|  | *w-;UAS-CsChrimson/+;VT002064-AD, dsxDBD/UAS-dsx^M^-RNAi* (*pC1^SS2^ > UAS-CsChrimson & UAS-dsx^M^-RNAi*) | GH-5-7d | 12 |
|  | *w-;UAS-CsChrimson/+;VT002064-AD, dsxDBD/+* (*pC1^SS2^ > UAS-CsChrimson*) | GH-5-7d | 12 |
|  | *w-;UAS-CsChrimson/+;VT002064-AD, dsxDBD/UAS-dsx^M^-RNAi* (*pC1^SS2^ > UAS-CsChrimson & UAS-dsx^M^-RNAi*) | GH-5-7d | 12 |
| Figure 6 | | | |
| B | *Canton-S* | GH-7d | 12 |
|  | *Canton-S* | SH-7d | 12 |
|  | *Canton-S* | GH-7d,  SH-7d | 12 |
| C | *Canton-S* | GH-14d | 12 |
|  | *Canton-S* | SH-14d | 12 |
|  | *Canton-S* | GH-14d,  SH-14d | 12 |
| E | *Canton-S* | GH-7d | 12 |
|  | *Canton-S* | SH-7d | 12 |
|  | *Canton-S* | GH-7d,  SH-7d | 12 |
| F | *Canton-S* | GH-14d | 12 |
|  | *Canton-S* | SH-14d | 12 |
|  | *Canton-S* | GH-14d,  SH-14d | 12 |
| G | *Canton-S* | GH-7d,  GH-14d | 12 |
|  | *Canton-S* | SH-7d,  SH-14d | 12 |
|  | *Canton-S* | GH-14d,  GH-21d | 12 |
|  | *Canton-S* | SH-14d,  SH-21d | 12 |
| H | *Canton-S* | GH-7d,  SH-14d | 12 |
|  | *Canton-S* | SH-7d,  GH-14d | 12 |
|  | *Canton-S* | GH-14d,  SH-21d | 12 |
|  | *Canton-S* | SH-14d,  GH-21d | 12 |
| Figure 1-figure supplement 1 | | | |
| A, B | *Canton-S* | SH-7d | 12 |
|  | *Canton-S* | SH-14d | 12 |
|  | *Canton-S* | GH-7d | 12 |
|  | *Canton-S* | GH-14d | 12 |
| Figure 3-figure supplement 1 | | | |
| A | *w+;;Or47b-GAL4/+* | GH-5-7d | 9 |
|  | *w+;;Or47b-GAL4/UAS-Or47b-RNAi* | GH-5-7d | 9 |
| B | *w+;;fru^GAL4^/+* | GH-5-7d | 9 |
|  | *w+;;fru^GAL4^/UAS-fruMi* | GH-5-7d | 9 |
| Figure 3-figure supplement 2 | | | |
| A | *UAS-myrGFP,QUAS-mtdtomato*(*3xHA*)*;trans-*  *Tango/+;Or47b-GAL4/+* | GH-14d, 18°C |  |
|  | *UAS-myrGFP,QUAS-mtdtomato*(*3xHA*)*;trans-*  *Tango/+;Or47b-GAL4/+* | SH-14d, 18°C |  |
| B | *UAS-myrGFP,QUAS-mtdtomato*(*3xHA*)*;trans-*  *Tango/+;Or47b-GAL4/+* | GH-14d, 18°C | 10 |
|  | *UAS-myrGFP,QUAS-mtdtomato*(*3xHA*)*;trans-*  *Tango/+;Or47b-GAL4/+* | SH-14d, 18°C | 8 |
| Figure 4-figure supplement 1 | | | |
| B | *w+;UAS-dTrpA1/+* (light, 22°C) | GH-5-7d | 18 |
|  | *w+;R15A01-AD/UAS-dTrpA1; R71G01-DBD/+* (*P1^a^ > UAS-dTrpA1*) (light, 22°C) | GH-5-7d | 18 |
|  | *w-;UAS-dTrpA1/+; VT002064-AD, dsxDBD/+* (*pC1^SS2^ > UAS-dTrpA1*) (light, 22°C) | GH-5-7d | 18 |
|  | *w+;UAS-dTrpA1/+* (dark, 22°C) | GH-5-7d | 18 |
|  | *w+;R15A01-AD/UAS-dTrpA1;R71G01-DBD/+* (*P1^a^ > UAS-dTrpA1*) (dark, 22°C) | GH-5-7d | 18 |
|  | *w-;UAS-dTrpA1/+;VT002064-AD, dsxDBD/+* (*pC1^SS2^ > UAS-dTrpA1*) (dark, 22°C) | GH-5-7d | 18 |
|  | *w+;UAS-dTrpA1/+* (light, 30°C) | GH-5-7d | 18 |
|  | *w+;R15A01-AD/UAS-dTrpA1;R71G01-DBD/+* (*P1^a^ > UAS-dTrpA1*) (light, 30°C) | GH-5-7d | 18 |
|  | *w-;UAS-dTrpA1/+;VT002064-AD, dsxDBD/+* (*pC1^SS2^ > UAS-dTrpA1*) (light, 30°C) | GH-5-7d | 18 |
|  | *w+;UAS-dTrpA1/+* (dark, 30°C) | GH-5-7d | 18 |
|  | *w+;R15A01-AD/UAS-dTrpA1;R71G01-DBD/+* (*P1^a^ > UAS-dTrpA1*) (dark, 30°C) | GH-5-7d | 18 |
|  | *w-;UAS-dTrpA1/+;VT002064-AD, dsxDBD/+* (*pC1^SS2^ > UAS-dTrpA1*) (dark, 30°C) | GH-5-7d | 18 |
| Figure 4-figure supplement 2 | | | |
| *retro-*Tango | *QUAS-mtdTomato*(*3xHA*)*;retro-Tango/R15A01-AD;UAS-EGFP/R71G01-DBD* (*P1^a^ > retro-Tango*) | GH-14-21d |  |
|  | *QUAS-mtdTomato*(*3xHA*)*;retro-Tango/+;UAS-EGFP/VT002064AD,dsxDBD* (*pC1^SS2^ > retro-Tango*) | GH-14-21d |  |
| GFP | *w-;R15A01-AD/+;R71G01-DBD/UAS-myrGFP* (*P1^a^ > GFP*) | GH-5-7d |  |
|  | *w-;;VT002064AD,dsxDBD/UAS-myrGFP* (*pC1^SS2^ > GFP*) | GH-5-7d |  |
| *trans-*Tango | *UAS-myrGFP,QUAS-mtdtomato*(*3xHA*)*;trans-Tango/R15A01-AD;R71G01-DBD/+* (*P1^a^ > trans-Tango*) | GH-14-21d |  |
|  | *UAS-myrGFP,QUAS-mtdtomato*(*3xHA*)*;trans-Tango/+;VT002064AD,dsxDBD/+* (*pC1^SS2^ > trans-Tango*) | GH-14-21d |  |
| Figure 6-figure supplement 1 | | | |
|  | *Canton-S* | GH-14d,  SH-14d | 12 |
| Figure 6-figure supplement 2 | | | |
| A | *Canton-S* | GH-14d | 12 |
|  | *Canton-S* | SH-14d | 12 |
|  | *Canton-S* | GH-14d,  SH-14d | 12 |
| B | *Canton-S* | GH-14d | n=15 events from 8-12 samples for each group |
|  | *Canton-S* | GH-14d,  SH-14d | n=15 events from 8-12 samples for each group |
| Figure 6-figure supplement 3 | | | |
| A | *w+;;UAS-Kir2.1/+* | GH-14d | 12 |
|  | *w+;;Or47b-GAL4/UAS-Kir2.1* | GH-14d | 12 |
|  | *w+;;Or47b-GAL4/UAS-Kir2.1 w+;;UAS-Kir2.1/+* | GH-14d | 12 |
|  | *w+;;VT002064AD,dsxDBD/ UAS-Kir2.1*(*pC1^SS2^ > UAS-Kir2.1*) | GH-14d | 12 |
|  | *w+;;VT002064AD, dsxDBD/ UAS-Kir2.1*(*pC1^SS2^ > UAS-Kir2.1*) *w+;;UAS-Kir2.1/+* | GH-14d | 12 |
| B | *w+;;UAS-Kir2.1/+* | GH-14d | 12 |
|  | *w+;;Or47b-GAL4/UAS-Kir2.1* | GH-14d | 12 |
|  | *w+;;Or47b-GAL4/UAS-Kir2.1 w+;;UAS-Kir2.1/+* | GH-14d | 12 |
|  | *w+;;VT002064AD,dsxDBD/ UAS-Kir2.1*(*pC1^SS2^ > UAS-Kir2.1*) | GH-14d | 12 |
|  | *w+;;VT002064AD, dsxDBD/ UAS-Kir2.1*(*pC1^SS2^ > UAS-Kir2.1*) *w+;;UAS-Kir2.1/+* | GH-14d | 12 |
