# Supplementary material for: Social experience shapes fighting strategies in Drosophila
Source: eLife. 2025 Oct 7;13:RP104212. doi: 10.7554/eLife.104212 (PMC12503488; doi:10.7554/eLife.104212)
Supplement: Supplementary file 2. [file elife-104212-supp2.docx]

**Supplementary File 2. Detailed comparison for territorial control and mating competition.**

| Territorial control assay | | | | |
| --- | --- | --- | --- | --- |
|  | **pairs** | **winning index** | **P value** | **conclusion** |
| Fig. 6B | G7a vs G7b | -10±30.15 | 0.275 | / |
|  | S7a vs S7b | 8.33±43.87 | 0.524 | / |
|  | G7 vs S7 | 21.66±50.06 | 0.1619 | / |
|  |  |  |  |  |
| Fig. 6C | G14a vs G14b | 8.33±49.33 | 0.5702 | / |
|  | S14a vs S14b | -3.33±59.60 | 0.8499 | / |
|  | G14 vsS14 | 48.33±65.76 | 0.0272 | G14>S14 |
|  |  |  |  |  |
| Mating competition assay | | | | |
|  | **pairs** | **copulation advance index** | **P value** | **conclusion** |
| Fig. 6E | G7a vs G7b | 3.33±22.29 | 0.6147 | / |
|  | S7a vs S7b | 3.33±14.35 | 0.4382 | / |
|  | G7 vs S7 | -5±17.32 | 0.3388 | / |
|  |  |  |  |  |
| Fig. 6F | G14a vs G14b | 0±19.07 | >0.9999 | / |
|  | S14a vs S14b | 0±17.06 | >0.9999 | / |
|  | G14 vs S14 | 26.67±32.29 | 0.0155 | G14>S14 |
|  |  |  |  |  |
| Fig. 6G | G7 vs G14 | 46.67±24.62 | <0.0001 | G7>G14 |
|  | S7 vs S14 | 43.33±25.35 | <0.0001 | S7>S14 |
|  | G14 vs G21 | 58.33±27.58 | <0.0001 | G14>G21 |
|  | S14 vs S21 | 51.67±24.80 | <0.0001 | S14>S21 |
|  |  |  |  |  |
| Fig. 6H | G7 vs S14 | 45±24.31 | <0.0001 | G7>S14 |
|  | S7 vs G14 | 18.33±21.67 | 0.0137 | S7>G14 |
|  | G14 vs S21 | 41.67±21.67 | <0.0001 | G14>S21 |
|  | S14 vs G21 | -25±28.44 | 0.0112 | S14<G21 |
| S21<S14<G21<G14 | | | | |
